# A qualitative study exploring patient shadowing as a method to improve patient-centred care: 10 principles for a new gold standard

**DOI:** 10.1093/intqhc/mzac018

**Published:** 2022-03-21

**Authors:** Joanna Goodrich, Damien Ridge, Tina Cartwright

**Affiliations:** Cicely Saunders Institute, King’s College London, Bessemer Road, London SE5 9PJ, UK; School of Social Sciences, University of Westminster, 115 New Cavendish Street, London W1W 6UW, UK; School of Social Sciences, University of Westminster, 115 New Cavendish Street, London W1W 6UW, UK; School of Social Sciences, University of Westminster, 115 New Cavendish Street, London W1W 6UW, UK

**Keywords:** quality improvement, patient-centred care, patient shadowing, patient experience, guidelines

## Abstract

**Background:**

In recent years, there has been an increased emphasis on patient experience as a dimension of quality in healthcare and subsequently a drive to understand care from the patient’s perspective. Patient shadowing is an approach that has been used in service improvement projects, but its potential as a quality improvement (QI) method has not been studied in practical and replicable detail.

**Objective:**

This new research aimed to produce clear guidance on patient shadowing for future Quality Improvement projects.

**Methods:**

Qualitative interviews were conducted with 20 clinical and non-clinical participants of a national QI programme in UK, which focused on improving the experience of patients at the end of life. All participants had shadowed patients. Data were analysed using a thematic analysis.

**Results:**

There were two broad themes: (i) The process of shadowing: how participants went about shadowing, adopting different approaches and making judgements about the care they observed and any challenges they had encountered. (ii) The impact of shadowing: on the engagement and motivation of those who shadowed and in terms of service changes to benefit patients and their families.

**Conclusion:**

The findings led to a new set of ‘gold standard’ principles to benefit both staff and patients where shadowing is used as a QI method. These, together with new guidance, will ensure that shadowing is conducted as a team exercise, that all those involved are more robustly prepared and supported and that its purpose as a method to improve patient experience will be better understood.

## Introduction

The Institute of Medicine’s internationally accepted definition of high-quality care has six elements: patient-centred, safe, effective, timely, efficient and equitable [1]. In UK, the three dimensions of quality are defined as patient experience, safety and effectiveness [2]. The relationship between elements of patient-centred care and improved experience has been established for some time [3–5] with corresponding quality improvement (QI) initiatives focusing on patient experience.

Most methods for assessing quality in healthcare strive to be objective in nature and there has been relatively little research about experiential approaches in QI, although these could be appropriate for programmes concerned with patient experience. One such example is patient shadowing, which involves accompanying patients wherever they are receiving care in order to observe their experiences of care. In healthcare training and education, experiential learning is used as a part of teaching about empathy [6] and mainly relates to training student health professionals, using simulation approaches where students experience being a patient through, for example, lying in a bed or spending a day in a wheelchair [7]. Immersive learning has been shown to increase understanding and connection to patients, and ‘eye-opening’ insights into their experience of living with illness, and empathy [8]. Recently, although the potential for QI of shadowing by researchers (who then relay their findings to healthcare staff) has been recognized [9], patient shadowing undertaken by healthcare staff, as a systematic approach to improvement, has not received the same attention or been studied thoroughly [10–16]. Likewise, the exact nature of the connection between shadowing and achieving improvement for patient experience has not been explored. This study has enabled us to make recommendations in the form of new guidance and principles about how best to conduct shadowing in order to be an effective QI method.

This study explored the experience of participants undertaking patient shadowing as part of a national QI programme. The premise of the programme was that there is a need to understand how patients experience a service, in order to improve their experience. Programme participants were thus required to adopt patient shadowing as a specific technique to ensure that the information they collected for their improvement projects described care from the patient’s perspective. The research study involved qualitative interviews with programme participants to gather detailed accounts of shadowing, and findings were explored qualitatively and reported previously [10]. The purpose of this paper is to focus on the practical guidance produced for future teams derived from the qualitative data. This guidance strengthens the patient shadowing method so that, carried out ethically and robustly, it can make a successful contribution to QI. A set of 10 clear principles for shadowing are presented for the first time.

### Study context: the Living Well Programme

Eighteen multidisciplinary healthcare teams (with up to five members each) from across UK took part in the Living Well Quality Improvement programme to improve the experience of patients at the end of life (in non-specialist palliative care services). The programme was funded by The Health Foundation with support from NHS England and led by The Point of Care Foundation, a not-for-profit organization. The programme followed a collaborative learning model, during which participants attended three learning events between July 2017 and April 2018. Participants were taught conventional QI methods [17], but first healthcare staff were required to shadow patients in their service, to inform their understanding of where to focus their improvement efforts. Participants in the programme were provided with a handbook and training by The Point of Care Foundation, which emphasized that shadowing is a purposeful and structured activity. The practical issues were set out, such as how to select patients to shadow, how to provide them with information, how to gain consent and how to conduct oneself when shadowing. Procedures to follow if participants noted anything of concern were set out clearly, and it was explained that being a shadower did not prevent them from helping the patient if needed; this is an important difference between shadowing as a research method and shadowing as a QI method.

Patients to be shadowed were selected at the discretion of clinical managers locally, and wherever possible, they were unknown to the shadower, although there were occasional exceptions. Patients included those being cared for at home or in a residential home, patients attending outpatients’ appointments and on a variety of inpatient hospital wards. Consent was gained from the patient and/or a family visitor. Participants in the QI programme could undertake shadowing through spending time with a patient, sitting with them or accompanying them if they were moving between locations and interacting with them if appropriate. Participants spent as little as half an hour once or several hours on a number of occasions, shadowing the same or different patients.

## Methods

The study design was qualitative, and semi-structured interviews were conducted with 20 programme participants, recruited from all 18 teams in the programme at face-to-face learning events and by email invitation. Maximum variation was sought in the sample [18]: participants had a range of professional backgrounds and work settings, including acute hospital wards, primary care, community and mental health services and nursing and care homes. The programme required project teams to be a mix of clinicians and non-clinicians and this was reflected in the sample. In this particular programme, the participants were all working with patients at the end of life but were not palliative care specialists. Interviews were conducted face to face at the participant’s workplace or by telephone or skype, depending on what was most convenient for each participant. The interview topic guide covered questions about the process of shadowing, what they had observed and asked for their reflections on the experience. In the end, they were asked for advice or tips for others in the future.

Interviews were transcribed verbatim and analysed using a thematic analysis (TA) as described by Braun and Clarke [19]. TA uses the basic building blocks of a qualitative analysis to assist the researcher to find a small number of important patterns/themes across a diverse sample that shed light on the research question. TA was chosen because it was important to analyse a broad range of experiences from a sample of individuals with diverse professional backgrounds, working in different end-of-life settings, enabling implications for practice to be drawn out and robust recommendations to be made for future teams, a key objective of the research. Data-derived codes were created inductively, and researcher-derived codes (latent codes) were created through identifying more implicit meanings in the data, drawing on the researcher’s professional experience, discussion with co-researchers and the research literature. A complete coding approach was taken, i.e. the transcripts were coded by hand line by line by the first author. Codes were grouped into themes and sub-themes as an iterative process with feedback from co-authors. A set of preliminary themes was then shared with the programme organizers specifically to check their relevance to the programme, as a key study objective was to produce guidance that the programme organizers would implement. Data were finally organized into 10 main themes, which included individual reflections on the emotional impact of shadowing (discussed elsewhere [10]). The themes relating to the practical aspects of the process of shadowing as a QI approach and the impact of patient shadowing (for staff and for service improvement) are presented in this paper. Reports produced by each project team documented the results achieved for patients through service changes [20], and these were compared with data from interviews, in order to produce the guidance for future programmes and principles for shadowing, as outlined in this paper.

The researcher had been employed by the organization that led the national programme but was not involved in this programme, being aware of the necessity of being objective when analysing the data and interpreting the findings [21].

## Results

There were two overall themes (Table 1) relevant to shadowing as a QI method: (i) ‘how’ shadowing was undertaken (the processes involved and the different approaches adopted), and (ii) the impact of shadowing (in terms of motivating programme participants and making improvements for patients). Quotes from participants to illustrate these themes are provided in (Table 2).

**Table 1:** Themes

Theme	Sub-theme
1. The process of shadowing	Styles and approaches Non-intervening
	Intervening
	Companion
	Making judgements and interpretations
	Challenges experienced by participants
2. Impact of shadowing	Staff motivation: ‘A thirst for quality improvement’
	Service improvement

**Table 2:** Illustrative quotes for themes

**Theme 1: The process of shadowing**	**Illustrative quotes**
Varied styles and approaches	
Non-intervening	‘Yeah it was quite strange, I didn’t think I’d get into the zone so quickly, and it was only within a couple of minutes and I was right there with her, and that surprised me…you take the carer or the nurse hat off and then you just become, you become part of them, and part of their environment’. (Healthcare assistant P10)
	‘Carers in a healthcare setting, they’re always doing and this [shadowing] is a shift into a more mindful, reflective state, and some people just never go there’. (Therapist, P16)
Intervening	‘I’m a person who likes to jump in and get stuck into things and be more active’. (Non-clinician, P20)
	‘The patient was trying to talk…so I opened the blind and let some light in, and I opened the door, you know, and turned the lights on’. (Therapy assistant, P11)
Companion	‘I hate to see or think of people being on their own and having no-one. So, although I would be shadowing, I might well be holding someone’s hand at the same time. I think it might have eased them, given them some comfort’. (Non-clinician, P2)
Making judgements	‘It was quite an upsetting experience at times, because you always think, or I always think, you know, of my family members. If that was my family member, what would I want and how would I want people to react, you know’. (Therapy assistant, P11)
Challenges	‘We used to have permission to sit and talk to patients…these days you’ve got to be up and doing [things]’. (Nurse, P3)
Theme 2: Impact of shadowing	Illustrative quotes
Staff motivation	‘I think it gives them [shadowers] a real, genuine insight into the lens of the patient. It gives them a thirst for quality improvements, to look at changes for improvements that they can engage in and make to improve patient experience……It’s made them think and understand and have the courage to take action’. (Nurse, P13)
	‘The people that have done it [shadowing] are so enthused by, it that I think we’ll start a shadowing programme for all sorts of things, and it does help with engagement’. (Doctor, P6)
Service improvement	‘It informs your decision-making and gives you a broader perspective. I don’t see how you can commission services without knowing what those services are and how they are being experienced’. (Non-clinician, commissioner, P8)
	‘I think you learn a lot about yourself as well as about your patients as well and how you can make improvements to their care and the whole family situation just by spending that time observing, seeing things’. (Community nurse, P17)

### The process of shadowing

#### Varied styles and approaches

Participants were given ground rules for shadowing as outlined above. However, the way they approached shadowing varied considerably and can be described within the following three categories:

- *Non-intervening*: The shadower observed without intervening and described imagining what it was like to be the patient. A medical consultant who shadowed a patient in a hospital side room (a room for one patient only) described the time spent ‘almost like meditation in a sense, of watching and listening and not doing anything, which is of course strange and a bit unusual’. A healthcare assistant in a care home described it as ‘getting into the zone’.

One participant who worked with patients with learning disabilities who could not speak explained that she felt her approach to shadowing came easily because she was used to watching and ‘tuning in’ to how patients were feeling.

- *Intervening:* The shadower intervened in the care of the patient, occasionally going beyond just helping the patient with something if needed. Shadowers with a more intervening style were more likely to have a nursing background. They tended to step in to care for the patient, either where they felt other staff did not have time or because it was instinctive for them to care for the patient because of their professional role; they found it hard to ‘do nothing’.
- *Companion*: The shadower acted in the role of companion for the patient. One shadower described deliberately chatting to the patient as they went from one outpatient appointment to another to check whether what she had noticed was aligned with what the patient was experiencing. Another held the patient’s hand while sitting with them. Those who saw themselves in the role of companion tended to be staff with non-clinical backgrounds and described shadowing as different from observation ‘you sort of accompany the patient. You’re with the patient…it’s with them rather than observation of them. I think it feels more shared’.

More than one of these approaches might be adopted in any shadowing session.

#### Making judgements

There was evidence of not only different approaches to shadowing but also different interpretations of what was observed. Participants’ ‘outlook on life’ or ‘lens’ determined their interpretation or judgement of what they saw when they shadowed. Personal experience of a similar situation with their own family could have a significant influence on how patients’ and families’ experience was perceived or interpreted. For example, one member of the staff explicitly said that she was seeking reassurance after a poor experience of seeing her grandmother die in hospital, ‘I almost wanted to see that it wasn’t like that…were things different?’. One clinician appeared to be checking, from a professional point of view, that care was adequate: ‘I reassured myself there was nothing to scare the horses’, meaning they saw nothing to worry about regarding care.

Generally, the participants seemed unaware that their observations were subjective, such as when making judgements based on their personal taste, such as passing comments on the type of music played on wards. One shadower described how she purposely identified with the families of the patients she was shadowing, through imagining they were her own family.

#### Challenges

The ‘acceptability’ of a method (how well it is received by participants) is an important consideration when asking healthcare staff to undertake QI work [22], and there were some minor practical challenges with shadowing. These included finding the time for shadowing, even though there was no prescribed amount of time for shadowing (it was suggested that participants should be pragmatic and shadow for as much time as they had available). Problems could arise if the purpose of shadowing was not explained clearly, particularly to colleagues; there was a sense that it could be misunderstood, particularly if it involved ostensibly ‘doing nothing’ sitting next to a patient who was in the bed. A clear explanation of its purpose allowed participants to adopt a shadowing role, rather than their usual role.

### Impact

There was evidence of a powerful personal impact on individuals who took part in shadowing. The majority of participants cited changes, such as increased knowledge or emotional engagement, which appeared to lead to greater motivation to make subsequent service changes intended to benefit patients and families.

#### Staff motivation: ‘A thirst for quality improvement’

Individuals spoke about the experience of shadowing in terms of increasing their knowledge and understanding of the care their patients received. Both clinical and non-clinical staff took part in shadowing, and the value of non-clinical staff participating was recognized as ‘bringing fresh eyes’ to the clinical setting: ‘We get very used to seeing certain things [and] you switch off to those things’. But clinical staff also acknowledged that shadowing allowed them to ‘step off the hamster wheel and get some headspace’. One participant gave the example of how she thought the ward was ‘busy, busy, busy’, but that when she spent an hour shadowing she realized that for the patients ‘nothing happens’; that there are long periods, particularly in single-side rooms when there is no interaction (clinical or otherwise) with members of staff and that patients can feel lonely and isolated. This example illustrates the QI concept of ‘work as imagined and work as done’ [23]: shadowing can be used to check that what clinicians think is happening matches the reality of processes, procedures and patient experience.

The emotional impact on the shadowers was clear for some. For example, participants described how the patient stayed with them in a way that differed from meeting patients under other circumstances and how they connected emotionally with particular patients or relatives they shadowed. Project teams anticipated possible emotional distress for shadowers and put support in place for them, but this was not taken up.

Frequently, the impact shadowing had on project participants was transformed into a desire for (and actual) change for patients that participants hoped was positive and a corresponding engagement with the improvement project. For example, a commissioner suggested that she now saw shadowing as essential for her role and guiding decision-making.

Shadowing could also impact on personal behaviour. One participant described how even if she was not sitting and shadowing she would now ‘always be watching things and looking out’. Many participants spoke of shadowing being a rewarding experience, and that it ‘reconnected’ them with patients and their own motivation to care: ‘It made a connection with why you’re doing it [being a doctor] in the first place’.

#### Service improvement

A key characteristic of the programme’s method was that participants in the project teams met after shadowing were able to identify together where change could be made, suggest ideas for improvement and then make the changes, in some cases immediately. The shadowing exercise encouraged the design of an ‘ideal experience’ for patients, plans for how to achieve this and identifying measures to monitor success. Improvements to the environment of care were suggested and made, both in terms of physical environment and processes, and to the way staff interacted with patients. These included reviewing the policy of putting patients at the end of life into side rooms, the provision of a special food menu, car parking and beds for family members and revising the approach to advance care planning conversations. In one case, further funding to continue the project was secured after the directors of the hospital heard about its success.

## Discussion

### Statement of principal findings

This study has provided increased insights into a practical approach to improving patient experience through understanding care from the patients’ perspective. It has shown how participants went about shadowing, adopting different approaches and making judgements about the care they observed, which were influenced by their personal experience or ‘lens’. Although there were some challenges, the experience of shadowing appeared to strengthen their motivation to provide good care and to introduce service changes, based on where shadowing had helped them to identify where improvements could be made to benefit their patients.

### Strengths and limitations

Box 1: New guidance for programme handbook and training

- Emphasis on preparing the participants beforehand so that they understand that they will bring their own experiences, personalities and professional ‘lens’ to how they interpret what they observe when shadowing (much as ethnographic researchers have to be reflexive).
- It is made clear that different approaches to shadowing are acceptable (as highlighted in this study) and training does not prescribe one approach over another (in other words ‘getting alongside the patient’ could be done by sitting quietly and thinking oneself into how the patient might be feeling, or it could be through a conversation with the patient).
- Preparation must include an acknowledgement that shadowing places staff in a situation with their patients, which is different from usual, and that it does not have to involve carrying out caring tasks and to be mindful of the purpose of shadowing.
- Preparation must include an emphasis on shadowing as a legitimate activity (‘proper work’) and it is essential that colleagues are prepared for the presence of shadowers by explaining its purpose and by sharing the written guidance for shadowers with everyone.
- Shadowing is positioned emphatically as a team activity. Support should be put in place for team members before they begin and team debrief after shadowing is essential. Shadowing in pairs is suggested and logging observations and reflections in a standard way amongst the team is important.
- The issue of what to do if poor care is seen can be addressed by discussing this as a team beforehand and agreeing a process to alert the appropriate member of staff should this happen.
- The importance of debriefing after shadowing with other team members is emphasized, so that observations and interpretations can be compared. For example, participants will have shadowed at different times of day, different patients and situations, and have had different emotional responses. Members of the project team will all bring valuable observations that could be interpreted in different ways, and a shared understanding can be reached. A reflexive approach is encouraged and is positive in terms of professional practice. The purpose of the debriefing is to reach a consensus about where to target improvements.

 

Box 2: Principles for shadowing

Overall principle: The shadowing exercise should be situated at all times within the context of its purpose to improve care for patients and their families, so that it does not become exploitative.

1. Ensure that shadowing is a focused exercise. Do not embark on shadowing unless you have the necessary agreement/support in place to make a number of improvements as part of the project.
2. The risk of vulnerable patients being exploited should always be avoided (for instance, by approaching them and their relatives sensitively to ask permission and explaining that they can decline to be shadowed).
3. Communication. Provide information about shadowing to patients and families beforehand and share documented improvements achieved with patients and families.
4. Gain patients’ consent to shadow beforehand or consent from families on their behalf (see guidance here https://www.pointofcarefoundation.org.uk/resource/patient-family-centred-care-toolkit/).
5. As an activity shadowing is not passive (or voyeuristic). In other words, you are free to interact with the patients and families and to take action if they need help of any kind.
6. Shadowing teams should be thoroughly prepared beforehand. They should feel confident, understand the purpose of shadowing and know what agreed procedure to follow should they observe anything that concerns them. Individuals should know and understand when it would be inappropriate to shadow. It should be emphasized that shadowers should step away if there are situations where it is inappropriate for a stranger to be present.
7. Colleagues should be informed beforehand what the shadowers are doing and why. Explain that it is a purposeful and structured activity for the benefit of patients and is not an inspection exercise. (Make the written guidance for shadowers available to colleagues.)
8. Emotional as well as practical support for those who might find shadowing difficult, or personally challenging, should be in place both before and after shadowing.
9. Shadowing should be a team activity. Teams should debrief after shadowing, compare notes, impressions and experiences. Share and discuss the implications of what shadowers have learned for changes for patients.
10. Be reflexive. Remember that interpretations of what has been observed (particularly, the interactions between people) will be subjective—influenced by your way of seeing things—so it is important to check with and discuss your interpretation with others.

In terms of the participants’ accounts, it is important to bear in mind that they are necessarily subjective and might conflict with patients’ perceptions; thus, it would be valuable to include research with patients and families to compare their experience of the shadowing approach. Nevertheless, much is discussed about the need to link the collection of patient experience data with improvement [24, 25], and approaches which do this are not often documented. It is rare to find examples of practical approaches that provide details, which enable improvement approaches to be replicated [21], and this study provides detailed insight into the shadowing approach, including challenges that may be encountered.

### Interpretation within the context of the wider literature

Historically, QI efforts have focused more on safety and effectiveness, with fewer resources put into improving the quality of patient-centred care [2]. This may be because of a dual challenge: how we know whether a service provides high-quality patient experience (relevant data may be lacking) and uncertainty about how to approach QI in this area (lack of evidence-based practical approaches) [26]. A core tenet of QI is that a change should be an improvement and, measurement challenges aside, without knowing what is important to patients and families in the first place, it is possible that a QI intervention may not focus on what will make a difference to patients. This study has addressed such issues.

### Implications for policy, practice and research

The study has provided evidence that makes clear the connection between shadowing and improvements for patient experience and shown shadowing to be a powerful and relatively inexpensive tool to add to and complement the range of approaches and techniques in QI. It has enabled us to make recommendations in the form of new guidance and principles about how best to conduct shadowing in order to be an effective QI method. Patient shadowing by healthcare staff was an under-researched area. However, it would be interesting to explore further certain practical aspects of shadowing, for example, the connection between ‘lens’ and shadowing style. It would be valuable, in terms of preparing teams for shadowing, to discover whether professional background or personal experience made it easier or more difficult to undertake shadowing successfully, for example, to explore systematically the difference between clinical and non-clinical participants’ experience and reactions, and whether clinical training is an advantage or disadvantage when making judgements about what is observed.

## New guidance

While the study findings demonstrated shadowing as a technique for bringing about potential improvements, participants’ accounts of the shadowing process highlighted that further guidance would be beneficial to ensure that shadowing was established as a robust and effective QI method. Suggested new guidance with a 2-fold focus on the importance of preparation and teamwork was produced by researchers (Box 1) and discussed and agreed with programme facilitators. This has been incorporated into the handbook and training provided for programme teams and is already being implemented.

A set of 10 principles were produced and agreed with The Point of Care Foundation (see Box 2) and is proposed as a ‘Gold Standard’ for shadowing. These principles are now distributed to all programme participants before they embark on their projects. These principles provide a robust foundation for ethical shadowing practice.

*Conclusions:* Patient shadowing sheds light on what needs to improve for patients, which other ways of collecting data, such as surveys, do not. Healthcare staff expressed surprise to see ‘what really happens’, which suggests that shadowing would be a valuable approach not only to improve patient experience but also patient safety. Moreover, it is an acceptable, indeed motivating approach, for the staff who take part. With clear guidance and standards, patient shadowing is a valuable QI method.

## Data sharing statement

The data that support the findings of this study are available on request from the corresponding author. The data are not publicly available due to privacy or ethical restrictions.
